# The impact of the Covid-19 pandemic and previous natural disasters on the mental health of healthcare workers in Puerto Rico

**DOI:** 10.1371/journal.pgph.0001784

**Published:** 2023-05-17

**Authors:** Ruthmarie Hernández-Torres, Marijulie Martínez Lozano, Irma Torres, Ernesto Rosario-Hernández, Alíxida Ramos-Pibernus, Ana Soto, Luisa Ortiz, Franco Mascayano, Eliut Rivera-Segarra

**Affiliations:** 1 Ponce Health Sciences University, Ponce, Puerto Rico; 2 Clinical and Translational Institute, University of Rochester, New York, New York, United States of America; 3 University of Puerto Rico, Medical Sciences Campus, San Juan, Puerto Rico; 4 NeoMed Center Inc., Gurabo, Puerto Rico; 5 Department of Epidemiology, Mailman School of Public Health, Columbia University, New York, New York, United States of America; 6 New York Psychiatric Institute, New York, New York, United States of America; University of Toronto Temerty Faculty of Medicine, CANADA

## Abstract

The objective of this study was to assess the impact of COVID-19 pandemic worries (e.g., fear of contagion) and previous exposure to natural disasters (e.g., hurricanes) on Healthcare Workers (HCWs) mental health in Puerto Rico. Participants completed a self-administered online survey including items on sociodemographic information, working conditions, fears, and worries about the COVID-19 pandemic, past natural disaster experiences, depressive symptoms, and resilience. Logistic regressions models were performed to explain the relationship between depressive symptomatology and COVID-19 experiences and worries. 40.9% (n = 107) of the sample were classified as having some level (mild to severe) of depressive symptomatology (PHQ-8 ≥5). Results reflect normal to high levels of psychological resilience (BRS; M = 3.7, SD = 0.7). A significant association was found between depressive symptomatology and psychological resilience (OR = 0.44, 95% CI: 0.25–0.77). The odds of having depressive symptomatology were almost five times higher (OR = 4.79, 95% CI: 1.71–13.44) among those who reported emotional coping difficulties during the pandemic after experiencing a natural disaster compared to those that did not, when adjusting for psychological resilience and residence region. Despite normal to high psychological resilience levels, HCWs who reported emotional coping difficulties due to previous disasters were at risk of developing depressive symptomatology. Results suggest interventions to address the mental health of HCWs could benefit from considering other individual and environmental factors beyond resilience. Findings could inform future interventions to promote HCWs’ well-being before, during, and after a natural disaster or pandemic outbreak.

## Introduction

The COVID-19 pandemic has strained health care systems worldwide. Healthcare workers (HCWs) have been particularly impacted by the system’s organizational challenges in responding to the COVID-19 pandemic, such as increased work demands, longer working hours, high caseloads, and increased potential exposure to COVID-19 infection [1]. As a result, research has identified HCWs as an at-risk population for mental health problems such as depression, anxiety, and sleep disturbances [2]. Additional stressors, such as worries of getting infected with COVID-19 or infecting their families, have also been linked to negative HCWs mental health and wellbeing [3]. Furthermore, the literature suggests associations between sociodemographic characteristics, such as age and gender, and the mental health impact of the COVID-19 pandemic among HCWs [4]. Those HCWs of older age and who identified as female were at greater risk of developing significant depressive symptoms [5].

Despite this, research has also reported that protective factors such as resilience can and often do emerge in the contexts of disasters, including the COVID-19 pandemic [6]. Unfortunately, most studies are from China, and few studies to date examine the mental health and resiliency of HCWs exposed to other increasing stressors such as natural disasters. Thus, there is still a need to understand better the pandemic’s impact on the mental health and wellbeing of HCWs exposed to other disasters in different contexts and regions, such as the Caribbean [2,7,8].

**Fig 1:**
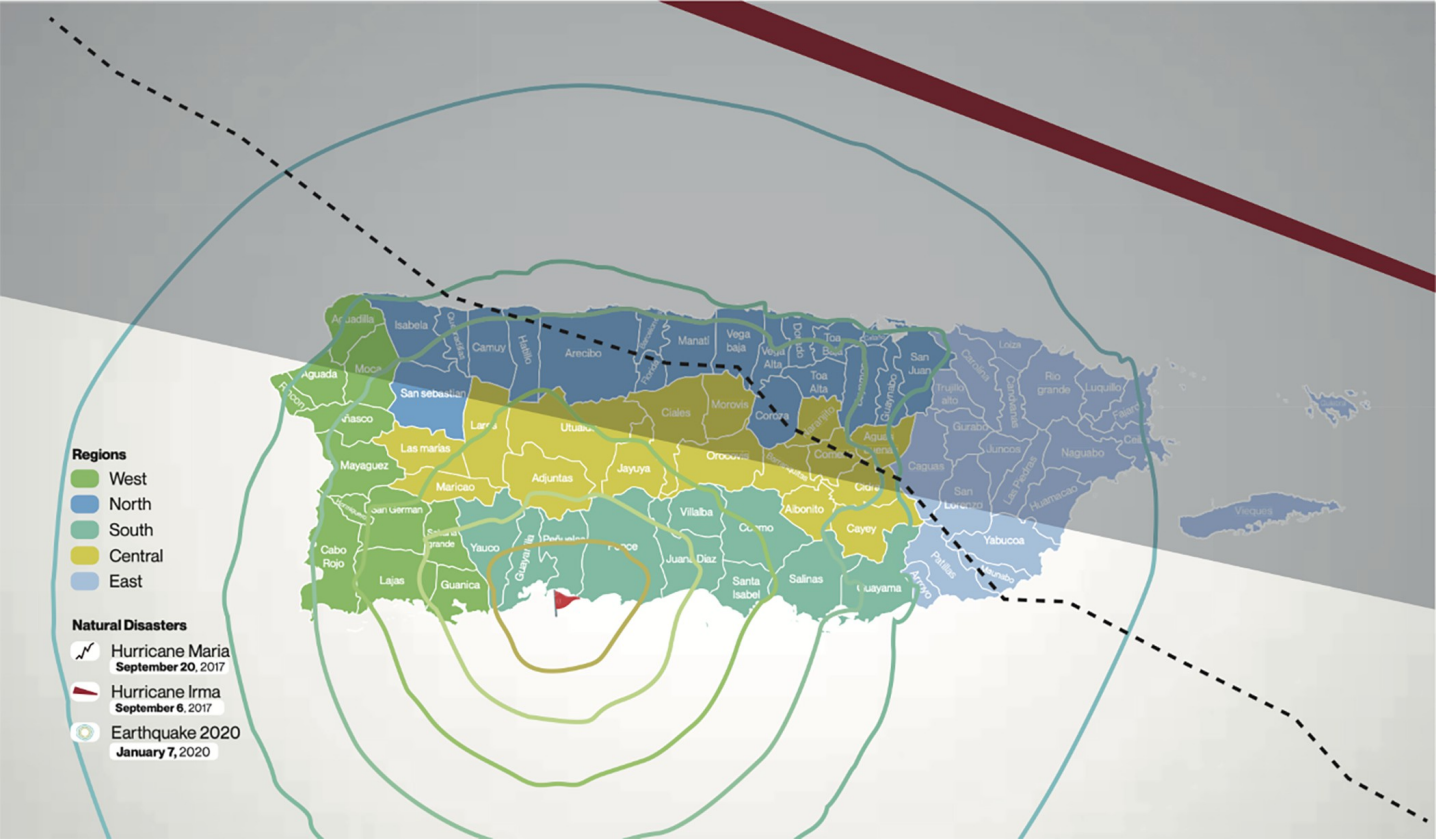
Puerto Rico and natural disasters impact by geographical region.

In Puerto Rico, the COVID-19 pandemic has magnified an ongoing social, political, economic, and health crisis. Puerto Rico is an archipelago in the Caribbean (Fig 1), where Spanish is the main language. It is a non-incorporated territory of the United States (US) subjected to a colonial status. The US government imposes significant restrictions on local government affairs, including healthcare funding, public health infrastructure, and disaster preparedness capacity [7,9]. Puerto Rico has experienced multiple waves of natural and social disasters during the last decade, including an economic recession, bankruptcy, and austerity measures’ imposition. In September 2017, two major hurricanes (Hurricane Irma and Hurricane María) impacted Puerto Rico in less than a two-week span. These extraordinary events caused a major humanitarian crisis with flooding, loss of homes, communications, blackouts, food shortage and access to potable water, increased chronic health conditions [8,10], and an estimated 2,975 excess mortality post-hurricane María [11,12]. In 2019, Puerto Rico’s governor was ousted due to a corruption scandal and mismanagement in the aftermath of hurricanes Irma and María. In January 2020, a swarm of earthquakes in the Southwestern region of the main island exacerbated an already unstable social and political landscape. It is within this context that the ongoing COVID-19 pandemic unfolded. This has led some researchers to posit that the threat to Puerto Rico during the COVID-19 pandemic lies principally in the context mentioned above, which has led to a normalization of exceptionality that has had a particularly detrimental impact on the healthcare system and, consequently, in HCWs [7,13,14].

This context has particularly impacted HCWs in Puerto Rico. For example, there is an ongoing increased migration of HCWs from the Caribbean archipelago to the mainland US, mainly due to insufficient healthcare infrastructure, severe funding cuts, and poor working conditions [15]. Recent studies evidence how following the impact of Hurricane María in Puerto Rico, HCWs experienced high levels of Post-Traumatic Stress Disorder, anxiety, and mental distress [16]. Furthermore, evidence from other essential workers in Puerto Rico shows a relationship between higher COVID-19-related work demands and increased depressive symptoms, suggesting the need to examine this further, especially among HCWs [17].

Despite this, research has also evidenced how community resilience has helped communities bounce back to address the emerging contextual needs in Puerto Rico after Hurricane María, mainly using renewable energy [18] and the provision of health care using innovative strategies [19]. However, little research has examined psychological resilience (defined as the individual’s capacity to bounce back or recover from stress) [20] during the COVID-19 pandemic among HCWs in Puerto Rico.

Thus, understanding the effects of the COVID-19 pandemic on the mental wellbeing of HCWs and the role of protective factors (i.e., psychological resilience) and sociodemographic characteristics in a context such as Puerto Rico, where compounding disasters co-occur, is critical to inform tailored healthcare management decision-making and intervention development. Considering this, the objective of this study was to examine the impact of the COVID-19 pandemic and previous exposure to natural disasters on the mental health and wellbeing of HCWs in Puerto Rico.

## Methods

This cross-sectional study is part of a more extensive prospective international study, the COVID-19 HEalth Care wOrkErS (HEROES) Study [21]. Inclusion criteria for potential participants included: 1) being over 21 years of age, 2) actively working at a healthcare facility that provides care to suspected or confirmed cases of COVID-19, and 3) having an internet connection to complete the online questionnaire. Recruitment was conducted in three stages. First, healthcare centers and professional organizations were identified and contacted by the research team. Those interested in participating directly shared the study’s invitation (containing general information about the research and a link to access the online survey). Secondly, study participants from the selected centers and organizations shared the study’s invitation to other potential participants from their respective professional networks via email and cellphone messages, among others. Finally, the research team also used media platforms (i.e., Facebook and radio) and social networks to share the study invitation to increase the study’s reach and sample.

Baseline data was collected between June 23, 2020, to October 31, 2020. Participants completed a self-administered online survey that included sociodemographic questions: age (years), gender (women, men, other), residence location (Northwest, Central, Southeast), educational level (Primary studies not completed, Primary studies, Secondary studies, Technical-professional training, University studies and Postgraduate studies [Master, doctorate, specialty]), and type of work (clinical and non-clinical). In addition, the baseline survey included questions regarding working conditions during the COVID-19 pandemic (e.g., In the last week, have you been around patients with suspected or confirmed COVID-19?).

*Worries*, *Experiences*, *and Concerns about Covid-19*. The team developed a questionnaire with eight questions about to Covid-19 worries, experiences, and concerns (e.g., In the last three months, how worried have you been about getting COVID-19). The questionnaire included two 5-point Likert scale items (worries) and six “Yes” or “No” items (experiences and concerns). For data analysis purposes, 5-point Likert scale items were regrouped as binaries and “Yes” and “No” questions were measured as independent items.

*Exposure to previous natural disasters*. The team also included questions to assess past experiences with natural disasters, hurricanes Irma and María, the 2020 earthquake sequence, and their perceived emotional coping strategies for regulating emotional reactions to a stressor (such as natural disasters and the pandemic). The natural disaster questions were categorized into three responses (i.e., Not at all, slightly to moderately, or very to extremely). The complete questionnaire can be found in Appendix 1. It also included two widely used measures validated in multiple languages (including Spanish which is the language in which the survey was deployed) examining depressive symptoms and resilience: Patient Health Questionnaire (PHQ-8) and the Brief Resilience Scale (BRS).

*Patient Health Questionnaire (PHQ-8)* is an 8-item self-administered version based on the Diagnostic and Statistical Manual of Mental Disorders (DSM-IV) criteria for major depression. Participants are required to answer whether they have experienced symptoms associated with depression within the previous two weeks (e.g., 1. *Little interest or pleasure in doing things*). It is answered using a 4-point Likert scale ranging from ‘not at all’ (0) to ‘almost every day’ (3). The Spanish Version of the PHQ-8 has been validated in Puerto Rico with a Cronbach’s alpha of .91 to .92 [22].

*Brief Resilience Scale (BRS)* is an 6-item scale measuring the individual’s psychological resilience (e.g., 1. *I tend to bounce back quickly after hard times*) [18]. It is comprised of a 5-point Likert scale ranging from ‘strongly disagree’ (1) to ‘strongly agree’ (5). The BRS is scored by reverse coding items 2, 4, and 6 and calculating the sum of all six items. This Spanish version of the BRS has shown good reliability scores with Spanish-speaking populations (Cronbach’s alpha = .83) [23].

### Ethics statement

This study was approved by the Ponce Health Sciences University (2004035367) and the Pan-American Health Organization (PAHOREC.0208.02) Institutional Review Boards. Potential participants accessed the online survey, which contained informed consent. Interested participants completed the online informed consent before engaging in the study.

### Data analysis

Statistical analyses were performed using Stata 16.1. Descriptive statistics were performed using an available case analysis. PHQ-8 and BRS scores were recoded for the bivariate analysis. For the PHQ-8, the five categories were recoded into two groups: (1) PHQ-8 <5 and (2) PHQ-8 ≥5 (Table 1). This cut-off point was selected because authors were interested in any depressive symptomatology, including low depressive symptoms (PHQ-8 ≥ 5–9) which is often missed if the clinical depression diagnosis cut-off score is used [24,25]. In addition, the lack of parametric distribution, small sample size, the variability observed in other samples regarding the cutoff selection (≥10 to ≥ 15), and the high percentage of participants reporting low (5–9) depressive symptomatology observed (27.5%) were key drivers for the team’s decision to use PHQ-8 ≥5 cut-off point [26]. The median was used as the cut-off point for the BRS to create two categories for resilience scores. This decision was made due to a lack of: 1) a defined cut-off point in the literature [20,23] and 2) normally distributed scores. Chi-square analysis was used to evaluate associations of COVID-19 concerns and experience (e.g., worry about getting infected, worry about infecting family, exposure to COVID-19 suspected/confirmed cases, managing emotions after natural disasters) and other covariates (e.g., gender, age, residence location, and resilience) with depression symptomatology (Table 2). Binomial logistic regression was performed to explain the relationship between depressive symptomatology and COVID-19 experiences and concerns (Table 3). Models were adjusted for covariates associated with depressive symptomatology (i.e., residence location and resilience).

**Table 1:** Demographic characteristics, resilience and depression symptomatology levels.

	N	%
**Age (years)**		
21–39	119	45.3
≥40	144	54.7
**Gender**		
Women	190	73.1
Men	70	26.9
**Residence location**		
Northwest	116	44.1
Central	40	15.2
Southeast	107	40.7
**Educational Level**		
Less than university studies	19	7.3
University studies	95	36.7
Post-graduate degree	145	56.0
**Type of work**		
Clinical	166	67.2
Non-clinical	81	32.7
**PHQ-8 score**	Median = 3.0	Q1 = 0, Q3 = 7.0
No symptomology (0–4)	155	59.2
Mild to severe symptomatology (5–24)	107	40.9
**BRS-Resilience**	Median = 3.7	Q1 = 3.2, Q3 = 4.0

The sample size will not always present a representation of the total recruited participants, and percentages will not always sum 100% due to small amounts of missing data.

**Table 2:** Association between covariates variables and depression symptomatology levels.

			p-value
	No symptomatology	Mild to severe symptomatology	
**Age (years)**			
21–39	63.9	36.1	0.158
≥40	55.2	44.8	
**Gender**			
Women	69.7	30.3	0.162
Men	77.6	22.4	
**Residence location**			
Northwest	50.4	49.6	<0.001
Central	85.0	15.0	
Southeast	58.9	41.1	
**BRS-Resilience**			
Below median (<3.7)	40.0	60.0	0.004
Above median (≥3.7)	60.4	39.6	
**Experiencing natural disasters made it more difficult to manage emotions during the pandemic**			
Not at all	68.0	32.0	0.001
Slightly to moderately	41.5	58.5	
Very to extremely	36.7	63.3	
**Experiencing natural disasters made it easier to manage emotions during the pandemic**			
Not at all	58.6	41.4	0.621
Slightly to moderately	50.5	49.5	
Very to extremely	47.9	52.1	
**Worry to get infected**			
None to some	62.4	37.6	0.014
A lot to very much	46.4	53.6	
**Worry to infect family**			
None to some	67.1	32.9	0.012
A lot to very much	49.7	50.3	
**Exposed to COVID-19 patient**			
No	61.5	38.5	0.023
Yes	44.4	55.6	
I don’t know	63.2	36.8	

**Table 3:** Binomial logistic regression results evaluating the depression symptomatology on COVID-19 experiences and concerns.

		OR crude	95% CI	OR adjusted*	95% CI
**Worry to get infected**					
None to some	Reference	1.00	-	1.00	-
A lot to very much		**1.92**	**1.14, 3.24**	**1.90**	**1.02, 3.56**
**Worry to infect family**					
None to some	Reference	1.00	-	1.00	-
A lot to very much		**2.07**	**1.17, 3.65**	1.56	0.81, 3.01
**Exposed to COVID-19 patient**					
No	Reference	1.00	-	1.00	-
Yes		**2.00**	**1.04, 3.83**	1.89	0.90, 3.99
I don’t know		0.93	0.48, 1.81	0.88	0.41, 1.90
**Experiencing natural disasters made it more difficult to manage emotions during the pandemic**					
None	Reference	1.00	-	1.00	-
A little bit to some		**3.00**	**1.59, 5.66**	**2.83**	**1.44, 5.57**
A lot to very much		**3.67**	**1.51, 8.91**	**4.79**	**1.71, 13.44**

*Residence location and resilience.

## Results

A detailed description of the study participants (n = 263) is presented in *Table 1*. The 40.9% (n = 107) of the sample reported having mild to severe depressive symptomatology (PHQ-8 ≥5). The median resilience score was 3.7 (Q1 = 3.2, Q3 = 4.0), reflecting normal resilience levels.

*Table 2* illustrates associations between covariates variables (i.e., age, gender, and residence location) and depression symptomatology. No association was found between age and gender with depressive symptomatology. Associations were found between residence location and depressive symptomatology (p = 0.001). A significant association was also found between resilience and depressive symptomatology (p = 0.004); experiencing natural disasters made it more difficult to manage emotions during the pandemic (p = 0.001), and worry about getting infected with COVID-19 (0.014) and worry to infect family (p = 0.012).

*Table 3* presents the impact of the four examined exposures (i.e., COVID-19-related worries and previous disaster experiences) on depressive symptomatology. Specifically, those who reported being highly worried about getting infected or infecting their families had more odds of having depressive symptoms than those who did not. Participants with a probable or confirmed case of COVID-19 exposure had twice the odds of having depressive symptoms compared with those who didn’t. After controlling for resilience and residence location, participants who reported being highly worried about getting infected had almost twice the odds (OR = 1.90, 95% CI: 1.02, 3.56) of depressive symptoms than those who reported not having or having low worrisome. However, when controlling for the same variables, no association was found between infecting family and exposure to COVID-19 patients with depressive symptomatology.

Participants with a resilience score of > 3.7 had less odds (OR = 0.44, 95% CI: 0.25, 0.77) of having depressive symptomology compared to those with a resilience score below the median (<3.7). Those participants who lived in the center (OR = 0.71, 95% CI: 0.42, 1.21) and in the southeast part of the island (OR = 0.18, 95% CI: 0.07, 0.46) had less odds of having depressive symptomology compared to those living in the northwest (Table 3).

After controlling for resilience and residence location, the odds of having depressive symptomatology were almost five times higher (OR = 4.79, 95% CI: 1.71–13.44) among those who reported higher emotional coping difficulties during the pandemic after experiencing a natural disaster compared to those that did not. Even participants that responded “a little bit” to some emotional coping problem during the pandemic after experiencing natural disasters had almost three times the odds (OR = 2.83, 95% CI: 1.44–5.57) of having depressive symptomatology compared to those that didn’t report difficulties.

## Discussion

HCWs experience a risk of mental health impacts during and after natural disasters, including hurricanes, earthquakes, and pandemics [27]. In this cross-sectional study, almost half the sample (40.9%) had some depressive symptomatology during the COVID-19 pandemic. These findings are consistent with previous research regarding the high levels of depressive symptomatology among HCWs during the COVID-19 pandemic in other geographical contexts [2]. We also found average levels of psychological resilience (85.6%) among HCWs despite the depressive symptomatology reported. Psychological resilience findings were consistent with earlier studies among HCWs in the US during the COVID-19 pandemic [3].

Interestingly, some of our results do not align with previous research findings, specifically regarding the role of psychological resilience as a potential protective factor for HCWs mental health and wellbeing. While some studies have documented negative correlations between mental health symptoms and resilience [3,16], in our study, after controlling for resilience, participants had higher odds of experiencing depressive symptomatology only if they had perceived emotional coping difficulties after a natural disaster. These where unexpected findings as researchers have also documented that having experienced an impact from a disaster is related to higher stress, not depressive symptomatology [8]. One potential explanation might be the so-called “resilience paradox,” which posits that more resilience might lead to more vulnerability in some contexts. It can reduce negative emotional responses to disasters and translate into less motivation and inducement to tolerate disparity and inequality [28]. This might be true in a context such as Puerto Rico, where social and political crises and continuing natural disasters have co- co-occurring. However, more research is needed to understand better this phenomenon’s role that might be related to the mental health and well-being of HCWs within the social, political, and cultural context of Puerto Rico.

Another important consideration is the inherent complex interaction between exposure to multiple natural disasters (COVID-19 pandemic, earthquakes and hurricanes) and depressive symptomatology. In our results, this complex interaction or syndemic, could be evidenced by the potential bidirectionality in the association between depressive symptomatology, COVID-19 worries, experiences and concerns, and difficulties managing emotions during previous natural disasters [29]. The Syndemics Framework posits that the interaction and co-presence of sequential diseases and the social and environmental factors can determine the process of health and disease [29]. Following this framework, our findings regarding depressive symptomatology among HCWs could be interpreted not only as the result of exposure to COVID-19 or multiple natural disasters independently, but rather by a long-term interaction and sequential exposure to natural disasters before and during the COVID-19 pandemic in addition to the social and environmental stressors previously mentioned (social, political, economic, and health crisis). This aligns with recent literature establishing the impact of the COVID-19 pandemic as a syndemic [30].

These findings have several implications, particularly for research and practice. In terms of research, our results suggest a need to understand better the role of resilience in the context of multiple disasters and among this population. Although we focused on psychological resilience, this is only one aspect of a multifactorial and dynamic concept. For example, recent research has begun to address community resilience in the context of Puerto Rico after Hurricane Maria [31]. However, community resilience’s role in the health and wellbeing of communities of practice such as HCWs remains an important and understudied topic, particularly in the context of multiple compounding disasters. In addition, research should examine perceived emotional coping in much more detail as we only examined it with one item. More evidence is still needed regarding specific emotional coping strategies (i.e., surface acting, attention deployment, or reflexivity) and their use before, during, and after disasters. For example, previous research has suggested that some common HCWs emotional coping strategies, such as surface acting (i.e., suppressing emotions) and attention deployment (i.e., focus on mechanical aspects of a given situation), although useful in some work-related situations, are linked to burnout [32]. Furthermore, the use of these strategies varies by gender, with some studies reporting higher use of dysfunctional emotional coping strategies (i.e., catastrophizing and rumination) among women HCWs [33]. However, there is still a limited understanding of their role in developing mental health pathologies in the context of disasters.

Finally, our findings suggest the need for a practice driven by contextually informed research and vice-versa, particularly regarding intervention development and implementation. For example, although psychological resilience can be an essential aspect of improving the health and well-being of HCWs in disasters, it cannot be assumed as an aprioristic protective factor for all populations in all contexts. Thus, our results suggest the need to foster mental health and well-being among HCWs in Puerto Rico by deploying interventions targeting other individual and environmental protective factors among workers (i.e., emotional regulation skills training) instead of solely resilience interventions. There are brief interventions developed and tested during the Covid-19 pandemic [34], although they will require tailored adaptations before deployment in Puerto Rico. Finally, based on our study findings, we echo Sovold and colleagues’ (2021) recommendations regarding the need to enhance HCWs positive working environments to improve their mental health and wellbeing [35]. As mentioned in the introduction, for the past decade, HCWs in Puerto Rico have faced multiple natural disasters, insufficient healthcare infrastructure, severe funding cuts, and poor working conditions, leading to an increased migration of HCWs. Police makers and organizations must address these needs to manage their mental health. Thus, emphasis must be on providing adequate staff, fair and timely pay, integrating HCWs in decision-making, and creating policies.

## Limitations

These findings should be interpreted considering some limitations. Due to a convenience sample, data does not represent the entire Puerto Rican HCWs population. The small sample size and cross-sectional design do not allow examining changes at different points of the COVID-19 pandemic. Emotional coping was not defined in the questionnaire. Thus, each participant could have interpreted something different from the other.

## Conclusion

Our study identified depressive symptomatology regarding normal resilience levels reported among HCWs in Puerto Rico. Also, despite normal levels of resilience, HCWs who reported emotional coping difficulties due to previous disasters (Hurricane Maria and earthquake sequence) were more at risk of developing depressive symptomatology. Although results cannot be generalizable to other contexts, they are significant as they can inform future work in the area. Findings show the need for further evidence evaluating the role of emotional coping strategies (i.e., surface acting, attention deployment, or reflexivity) and their use before, during, and after natural disasters among HCWs. In addition, our study confirms the need for interventions to target other individual and environmental protective and threatening factors among HCWs (i.e., emotional regulation skills training) instead of solely resilience interventions. Intervention development must emphasize providing adequate staff, fair and timely pay, integration of HCWs in decision-making, and creating policies, considering their particular geographical and political context.
